# Impact of prescription isodose level and collimator selection on dose homogeneity and plan quality in robotic radiosurgery

**DOI:** 10.1007/s00066-021-01872-4

**Published:** 2021-12-09

**Authors:** Alexandra Hellerbach, Markus Eichner, Daniel Rueß, Klaus Luyken, Mauritius Hoevels, Michael Judge, Christian Baues, Maximilian Ruge, Martin Kocher, Harald Treuer

**Affiliations:** 1 Faculty of Medicine and University Hospital Cologne, Department of Stereotaxy and Functional Neurosurgery, University of Cologne, Kerpener Straße 62, 50937 Cologne, Germany; 2 Faculty of Medicine and University Hospital Cologne, Institute of Radiation Oncology, University of Cologne, Cologne, Germany

**Keywords:** Cyberknife, Dose gradient, Homogeneity index, Collimator influence, Treatment time

## Abstract

**Purpose:**

In stereotactic radiosurgery (SRS), prescription isodoses and resulting dose homogeneities vary widely across different platforms and clinical entities. Our goal was to investigate the physical limitations of generating dose distributions with an intended level of homogeneity in robotic SRS.

**Methods:**

Treatment plans for non-isocentric irradiation of 4 spherical phantom targets (volume 0.27–7.70 ml) and 4 clinical targets (volume 0.50–5.70 ml) were calculated using Sequential (phantom) or VOLO^TM^ (clinical) optimizers (Accuray, Sunnyvale, CA, USA). Dose conformity, volume of 12 Gy isodose (V12Gy) as a measure for dose gradient, and treatment time were recorded for different prescribed isodose levels (PILs) and collimator settings. In addition, isocentric irradiation of phantom targets was examined, with dose homogeneity modified by using different collimator sizes.

**Results:**

Dose conformity was generally high (nCI ≤ 1.25) and varied little with PIL. For all targets and collimator sets, V12Gy was highest for PIL ≥ 80% and lowest for PIL ≤ 65%. The impact of PIL on V12Gy was highest for isocentric irradiation and lowest for clinical targets (VOLO^TM^ optimization). The variability of V12Gy as a function of collimator selection was significantly higher than that of PIL. V12Gy and treatment time were negatively correlated. Plans utilizing a single collimator with a diameter in the range of 70–80% of the target diameter were fastest, but showed the strongest dependence on PIL.

**Conclusion:**

Inhomogeneous dose distributions with PIL ≤ 70% can be used to minimize dose to normal tissue. PIL ≥ 90% is associated with a marked and significant increase in off-target dose exposure. Careful selection of collimators during planning is even more important.

**Supplementary Information:**

The online version of this article (10.1007/s00066-021-01872-4) contains supplementary material, which is available to authorized users.

## Introduction

Stereotactic radiosurgery (SRS) is a well-recognized and successful treatment modality for patients with malignant or benign intracranial pathologies [1–6]. Its use and importance are increasing as modern imaging techniques allow early detection of small brain lesions [7] and as recent advances in systemic therapy often lead to the need for further radiotherapy of, for example, new brain metastases and meningeomas, which is generally possible with SRS [8].

SRS differs in several aspects from standard radiation therapy, especially with regard to fractionation, target size, safety margins and dose prescription [9–12]. In SRS, very high doses are applied to the target volume in one or only a few fractions, which imposes strict requirements on minimizing the dose to normal brain tissue. This is achieved by minimizing the safety margins using stereotactic head fixation or inter- and intrafractional imaging, by conforming the high doses to the shape of the target and by a steep dose fall-off at the periphery of the target volume. To prevent the dose from spreading to normal tissue, high demands are placed on the accuracy of the beam guidance and the collimation of the radiation field. In many SRS systems, a set of cones with fixed circular field sizes are used for this purpose. With these circular collimators, dose conformation to the target volume can either be achieved by superposition of multiple isocentric shots (‘sphere packing’) or by application of a non-isocentric beam array adapted to the target shape [13]. The use of sphere packing inevitably leads to rather inhomogeneous dose distributions with cold and hot spots, whereas non-isocentric irradiation, e.g., with a robot-guided LINAC, has the potential to achieve more homogeneous dose distribution within the target. Therefore, different techniques for SRS such as Gamma Knife, Cyberknife and LINAC usually use different isodose prescription regimens [14]. Whether a homogeneous or an inhomogeneous dose distribution within the target is desired is controversial and depends on the clinical situation. For targets such as brain metastases, dose escalation within the target appears advantageous since these do not contain healthy tissue. In contrast, a more homogeneous dose distribution appears suitable for targets such as vestibular schwannoma or pituitary adenoma, as healthy tissue may also be included in the target volume [12, 15–18].

The aim of this theoretical study was to investigate the physical constraints for the intended generation of homogeneous or inhomogeneous dose distributions in robotic radiosurgery. In particular, the influence of the prescription isodose level (PIL) and collimator selection on dose conformity, volume of 12 Gy isodose (V12Gy) as a measure for dose gradient and treatment time were investigated.

The influence of the PIL on dose gradient to surrounding tissue has already been analyzed for different SRS platforms [19–23], although there was only one publication related to robotic stereotactic radiosurgery [22]. Contrary to that study we focused our analysis on the physical principles by eliminating the influence of planning skills and by including the influence of collimator sizes. In our study, we perform a systematic analysis including an ideal isotropic model, isotropic as well as non-isotropic phantom plans, and clinical cases to evaluate the impact of dose homogeneity and the effects of different collimator choices on SRS plan quality using a large amount of single-target SRS plans.

## Materials and methods

### Ethics statement

This retrospective study was approved by the local ethics committee of the University Hospital of Cologne (file number 16-476).

### Isocentric phantom irradiation

Dose–volume histograms (DVHs) for an artificial spherical target with a diameter of 8 mm (0.27 ml) and a shell with an outer diameter of 28 mm were calculated assuming an isotropic model. Within this model, the focal dose distribution *D* delivered by a conical collimator is given by

1 $$D\left(R\right)=2\pi \int _{0}^{\pi /2}d\theta sin\theta OCR\left(Rsin\theta \right)$$

where *OCR* is the measured beam profile of the collimator and *R* is the radial distance from the isocenter [24, 25]. The dose homogeneity, often described by a homogeneity index HI (HI = 100/PIL), was modified by using different collimator sizes. Collimators with diameters of 5.0, 7.5, 10.0, and 12.5 mm were used and a peripheral dose of 20 Gy was applied.

To verify the assumptions made in the isotropic model, dose distributions of isocentric beam sets were calculated using the Cyberknife planning software Precision 2.0.1.1 (Accuray, Sunnyvale, CA, USA) for a spherical phantom target with a diameter of 8 mm (0.27 ml). Due to the limited number of beam directions (the isocentric optimization algorithm for the full path head used allows a maximum of 133 beam directions), a slightly non-isotropic dose distribution emerged. The same collimators as in the isotropic model were used for the optimization, and equally weighted beams (non-conformal beam weights) and conformal beam weights (weighting of the beams to adapt the irradiation field to counteract non-isotropy) were applied. Around the phantom’s target a shell with an outer diameter of 28 mm (corresponds to 10 mm distance to the target surface) was generated to analyze the influence of PIL and collimator diameter on dose gradient using dose–volume histograms of the target and the shell. As in the isotropic model, a peripheral dose of 20 Gy was applied.

### Non-isocentric phantom irradiation

Using the non-isocentric irradiation technique of the Cyberknife, the influence of PIL and collimator selection on plan quality was evaluated. For this purpose, 4 spherical targets with volumes of 0.27, 0.55, 2.16 and 7.77 ml were generated in the center of a virtual spherical CT phantom. The Sequential optimization algorithm of the Cyberknife [26] was used to calculate treatment plans for different sets of collimators (including the Iris collimator [27]) and for different PILs (40–90% in steps of 10%). The same Sequential optimization scripts was used for all plans, consisting of (i) optimizing coverage of the target volume with the prescribed dose, (ii) optimizing conformity by sequentially minimizing the dose in 4 shell structures, and (iii) minimizing the total monitor units (MU) of the plan. Shells with distances of 3, 7, 12, and 20 mm to the target surface were used for optimization, with each shell having a thickness of 1 mm. Identical optimization objectives and weights were used except for the maximum dose, which was manually adjusted accordingly in each case to vary the PIL. For better comparability and in order to keep the influence of the coverage on the plan results to a minimum, uniform coverage were aimed for all plans. Using the Sequential optimization, full coverage of the targets could be achieved for all spherical phantoms by normalizing the minimum target dose to 20 Gy.

### Non-isocentric irradiation in clinical cases

Furthermore, 4 clinical targets with volumes of 0.50, 0.92, 2.01, and 5.68 ml (supplementary material, Figure S 1) were selected from our patient archive. A sample was chosen that was representative of the spectrum of brain metastases treated with Cyberknife and comparable in size to the phantom targets. The Cyberknife’s VOLO^TM^ optimizer [28] was used to calculate treatment plans for different sets of collimators including the Iris collimator as well as for different PILs (50–90% in steps of 10% and ≥ 90%). The smallest field size of the Iris collimator was 7.5 mm according to the manufacturer’s restriction for clinical applications. Similar parameter settings and optimization aims and weights were used as for the applied clinical plans, except for the maximum dose, which was adjusted accordingly to vary the PIL. In detail, 9 shells with distances of 2, 5, 10, 15, 20, 30, 50, 70, and 100 mm to the target surface were used for optimization, the total MU penalty value ranged between 0.5 and 0.7 and the maximum MU per beam was set to 150 for three clinical targets and 120 for one target. In all plans, the marginal dose was adjusted to 20 Gy and to an intended coverage of 99.5–99.8% as applied in the clinical plan. For example, with a prescription of 20 Gy at 65% PIL, the planner aims to achieve a maximum dose of 30.77 Gy, while a volume of 99.5% should receive 20 Gy.

### Analysis of plan quality

The influence of PIL and collimator selection on plan quality was analyzed using the conformity index nCI (reciprocal of the conformation number [29]), V12Gy, and the treatment time. Other metrics for the dose gradient were the volume of the 4, 8, and 10 Gy isodose outside the target (V4Gy, V8Gy, V10Gy), the gradient index GI [30], and the gradient measure GM [31]. The used quality indices were defined as followed:

2 $$nCI=\frac{PTV\cdot PIV}{\left(\mathrm{PTV}_{\mathrm{PIV}}\right)^{2}}$$

3 $$GI=\frac{\mathrm{PIV}_{50\% }}{PIV}$$

4 $$GM=\left(\frac{3}{4\pi }\right)^{1/3}\cdot \left[{\mathrm{PIV}_{50\% }}^{1/3}-\mathrm{PIV}^{1/3}\right]$$

where PTV is the planning target volume, PIV the prescription isodose volume, PTV_PIV_ the planning target volume that is covered by the prescription isodose volume, and PIV_50%_ the volume that is encompassed by half of the prescription isodose. In addition to treatment time, the total number of monitor units (TotalMU) and the number of beams (nBeams) were recorded.

Two different optimizers are used in this study to examine whether the results are independent of the selected optimizer.

### Statistics

The statistical analysis was performed using scatter plots and box plots. The Wilcoxon rank-sum test was used to assess the differences between V12Gy after grouping into 4 or 5 classes of the prescribed isodose level. The statistical dependence of V12Gy with the other gradient measures (V4Gy, V8Gy, V12Gy, GI, GM) and of the treatment time with TotalMU and nBeams was evaluated using Spearman’s rank correlation coefficient ρ where *p* ≤ 0.05 was used as the significance level. Furthermore, we assumed a linear model with interaction terms for the relationship between the dependent variable V12Gy and PTV, PIL and treatment time (TT):

5 $$V12Gy=\beta _{0}+\beta _{1}\cdot PTV+\beta _{2}\cdot PTV\cdot \left(PIL-50\% \right)+\beta _{3}\cdot PTV\cdot \left(TT-30\min \right)$$

The results were expressed as ranges or mean ± standard deviation. Statistical evaluation was performed with R v3.6.3 (https://www.r-project.org).

## Results and discussion

### Isocentric phantom irradiation

**Fig. 1:**
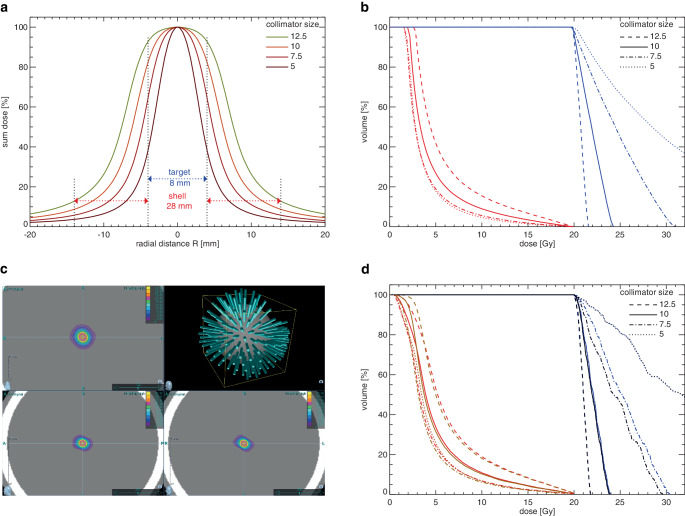
**a** Theoretical dose distributions and **b** resulting dose–volume histograms for a spherical phantom target with a diameter of 8 mm (0.27 ml, *blue*) and a shell with an outer diameter of 28 mm (*red*). **c** Dose planning for an isocentric beam set with conformal beam weights at the Cyberknife and **d** resulting dose–volume histograms for two beam weight options for a 0.27 ml spherical phantom target (conformal beam weights: *black*, non-conformal beam weights: *blue*) and a shell with an outer diameter of 28 mm (conformal beam weights: *brown*, non-conformal beam weights: *red*)

Based on measured beam profiles and equation (1), the isotropic model was used to calculate the theoretically achievable dose distributions that would result from isotropic, isocentric irradiation of a spherical target. Fig. 1a shows the resulting dose distributions of the 4 smallest collimators of the Cyberknife. Using these collimators for irradiating a spherical target with a diameter of 8 mm with a peripheral dose of 20 Gy resulted in maximum doses of 52.1, 31.0, 24.3, and 21.6 Gy for the collimator sizes 5.0, 7.5, 10.0, and 12.5 mm (Fig. 1b). The corresponding dose–volume histograms (DVHs) for dose prescriptions of 20 Gy at the 38%, 65%, 82%, and 93% isodose levels clearly show the dose-sparing effect outside the target volume with simultaneous dose escalation within the target for the lower PILs associated with the smaller collimator sizes (Fig. 1b). The isotropic model was compared with dose distributions of isocentric beam sets calculated at the Cyberknife using the same target size and collimators (Fig. 1c). Application of non-conformal and conformal beam weights resulted in very similar DVHs (Fig. 1d). As in the isotropic model, the dose-saving effect in the surrounding shell and the dose escalation within the target volume was again clearly visible at the lower PIL. For example, dose planning for an isocentric beam set with conformal beam weights resulted in maximum doses of 55.9, 29.6, 23.8, and 21.8 Gy for the collimator sizes 5.0, 7.5, 10.0, and 12.5 mm, while V12Gy was 1.7 and 3.1 times higher at isodose levels of 83% and 93%, respectively, than at levels ≤ 65%. Further results of the two beam weight options can be found in the supplementary material (Table S 1).

The results confirm that with isocentric irradiation, the dose-sparing effect at low PIL is mainly due to the size and shape of the beam profiles.

### Non-isocentric irradiation in phantom and clinical targets

**Fig. 2.1:**
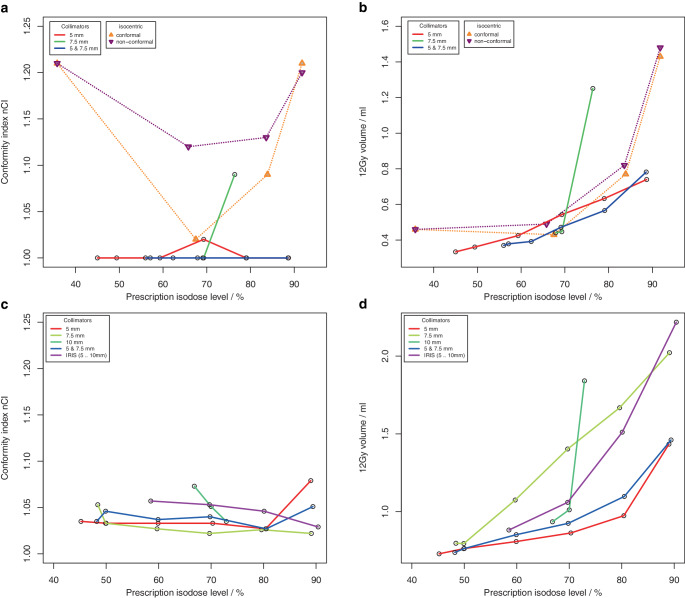
**a**, **c** Conformity index nCI and **b**, **d** volume of the 12 Gy isodose for 4 spherical phantom targets with volume **a**, **b** 0.27 ml, **c**, **d** 0.55 ml as a function of the achieved prescription isodose level. **a**, **b** Results of the isocentric planning are also shown

**Fig. 2.2:**
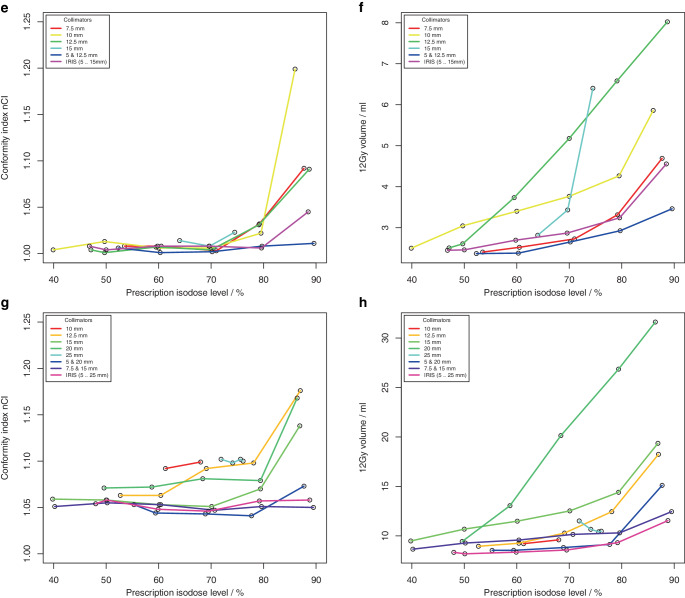
**e**, **g** Conformity index (nCI) and **f**, **h** volume of the 12 Gy isodose for 4 spherical phantom targets with volume **e**, **f** 2.16 ml and **g**, **h** 7.77 ml as a function of the achieved prescription isodose level

**Fig. 3.1:**
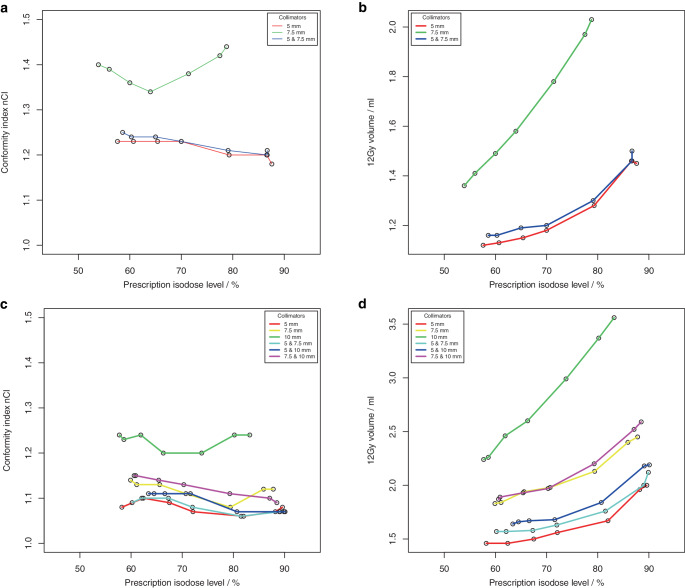
**a**, **c** Conformity index nCI and **b**, **d** volume of 12 Gy isodose for 4 clinical targets (single metastases) with volumes **a**, **b** 0.50 ml and **c**, **d** 0.92 ml, as a function of the achieved prescription isodose level

**Fig. 3.2:**
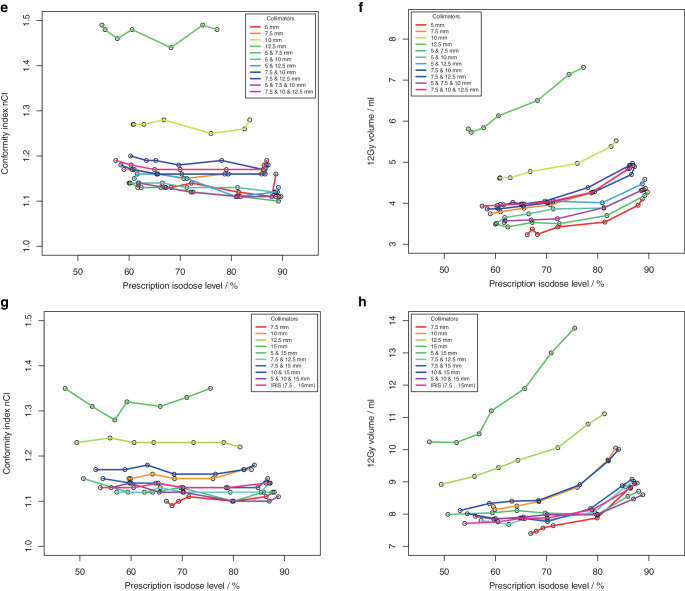
**e**, **g** Conformity index nCI and **f**, **h** volume of 12 Gy isodose for 4 clinical targets (single metastases) with volumes **e**, **f** 2.01 ml and **g**, **h** 5.68 ml as a function of the achieved prescription isodose level

The ability to generate dose distributions with high conformity and steep dose gradient with an intended homogeneity using non-isocentric irradiation was investigated for 4 spherical phantom targets and for 4 clinical targets (supplementary material, Figure S 1). For the phantom targets, a total of 110 treatment plans for 22 different collimator sets were computed using Sequential optimization (supplementary material Table S 2). For the clinical targets, a total of 210 treatment plans for 30 different collimator sets were calculated using VOLO^TM^ optimization (supplementary material, Table S 3). Fig. 2 (Fig. 2.1 + Fig. 2.2, phantom targets) and Fig. 3 (Fig. 3.1 + Fig. 3.2, clinical targets) show scatter plots of the resulting dose conformity index nCI and V12Gy as a function of the achieved PIL (box plots in supplementary material, Figures S 2 and S 3).

The achieved isodose levels were close to the intended target isodose levels, the deviation amounted to 0.1% ± 2.6% (range: −7.1% to 8.4%) for Sequential optimization and −0.5% ± 5.5% (range: −19.1% to 19.3%) for VOLO^TM^. Although for spherical targets only a limited range of PILs could be achieved with some collimator sets, the non-isocentric technique of the Cyberknife generally allowed the generation of plans with a PIL in the range of about 50–90%. In contrast to isocentric planning, the achieved PIL was no longer dependent on collimator size; however, target size was a limiting factor in achieving low PILs. Inhomogeneous irradiation, i.e., low PILs could only be achieved if the size of one collimator in a used collimator set was smaller than the target size.

The quality of the plans and the treatment time varied greatly among the different collimator sets and prescription isodose levels. While conformity was generally high (nCI ≤ 1.25) and showed very little dependence on the PIL in the phantom study using Sequential optimization, several outlier cases were observed for the clinical targets and the VOLO^TM^ optimizer associated with the collimator. Concerning the dose gradient of the plans, a much greater variability was observed, and there was a clear dependence of V12Gy on the PIL. In all targets and collimator sets, the lowest V12Gy was observed at isodose levels ≤ 65% and the highest V12Gy at isodose levels ≥ 80%. But generally, the variability of V12Gy as a function of collimator selection was higher than the increase of V12Gy with the prescribed isodose, and except for the smallest target, there was always at least one collimator that showed a very pronounced dependence of V12Gy on isodose level. Field sizes of these collimators were all in the range of 70–80% of the target diameter. Excluding these outlier curves, for spherical targets, V12Gy was on average a factor of 1.7 to 2.2 larger at isodoses > 85% than at isodose levels ≤ 55%, and for clinical targets, V12Gy was on average a factor of 1.1 to 1.3 larger at isodoses > 85% than at isodose levels ≤ 65%. For the smallest target, V12Gy was increased only by a factor of 2.2 and not by 3.1 as in isocentric irradiation, demonstrating the superiority of non-isocentric beam superposition in producing homogeneous dose distributions. However, this superiority only applied to collimators whose field size was not in the range of 70–80% of the target diameter, or to combinations of collimators. If the collimator was only slightly smaller than the target, the optimizer obviously needs to push the beams to the edge of the target and even beyond to avoid dose overlap in the center. Therefore, for these collimators, dose homogeneity could only be achieved by compromising the dose gradient.

A similar dependence of the dose gradient on the prescribed isodose level as found here was observed for all SRS platforms including robotic SRS [19–23]. Generally, V12Gy is lowest for prescriptions isodoses ≤ 65% and is increased by some 10% to 30% or more at 80% or 90%. By ruling out operator dependence in our study, we were able to show that such dependence already arises for purely physical reasons and therefore cannot be avoided. However, our results also show that the impact of collimator selection on plan quality is even larger than that of the PIL, emphasizing the need for experienced treatment planners.

**Fig. 4:**
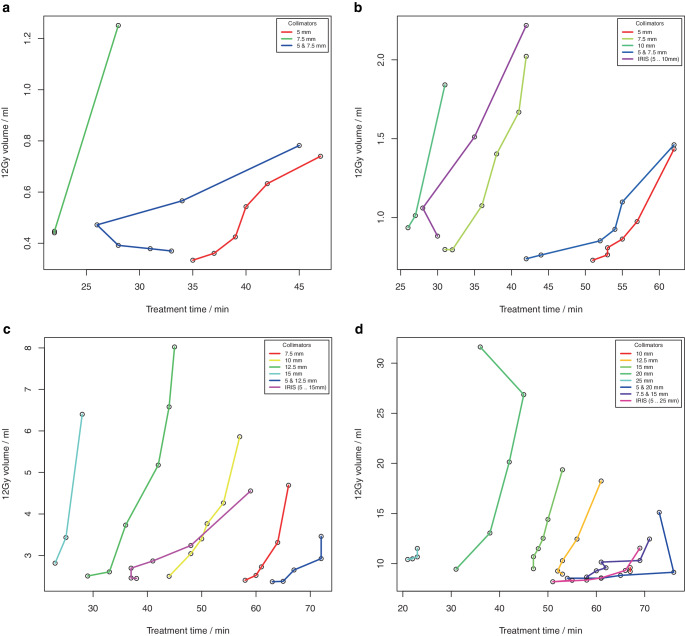
Volume of the 12 Gy isodose for four spherical targets with volumes **a** 0.27 ml, **b** 0.55 ml, **c** 2.16 ml, and **d** 7.77 ml as a function of the treatment time. Within one collimator set V12Gy increased with increasing prescribed isodose level (PIL)

**Fig. 5:**
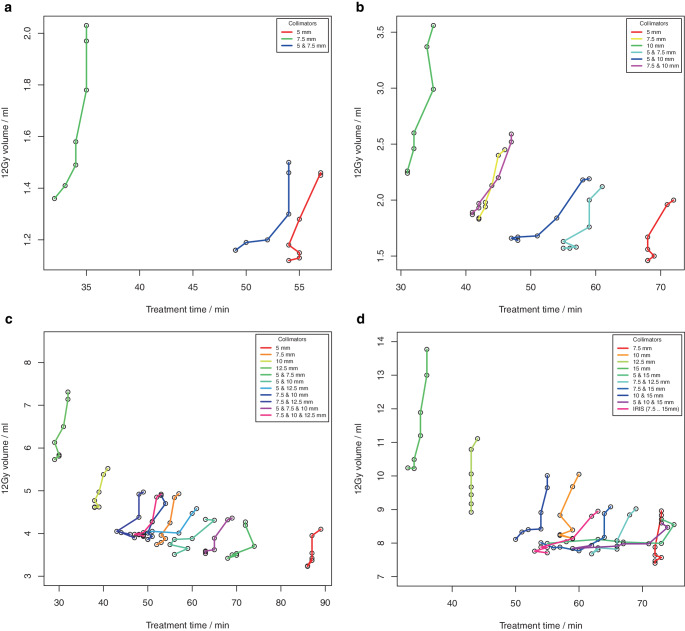
Volume of the 12 Gy isodose for four single metastases with volume of **a** 0.50 ml, **b** 0.92 ml, **c** 2.01 ml and **d** 5.68 ml as a function of the treatment time. Within one collimator set V12Gy increased with increasing prescribed isodose level (PIL)

Treatment time generally increased with increasing PIL, but again the main factor was collimator selection (supplementary material, Figures S4 and S5). There was a negative correlation of V12Gy with treatment time depending on collimator selection (Figs. 4 and 5). Within a collimator set, V12Gy increased with increasing PIL and thus with increasing treatment time (supplementary material, Figures S4 and S5), but when comparing different collimator settings, V12Gy decreased with increasing treatment time. The dependence of V12Gy on PIL and treatment time could be described by a linear model (Eq. 5), where R^2^ = 0.96 for clinical targets and R^2^ = 0.82 for phantom targets. Results of the linear regression parameters showed an increase of V12Gy with increasing target volume and PIL and a negative correlation with treatment time (Table 1), where the treatment time can be used as an indicator for collimator selection. Plans with steep dose gradients (low V12Gy values) could be achieved with PILs up to 80%, but the more homogeneous the selected PIL was, the greater the impact of collimator selection. Low V12Gy values with homogeneous PIL could only be achieved at large treatment times (Figs. 4 and 5).

**Table 1:** Results of the regression coefficients β_i_ for estimating the relationship between V12Gy and planning target volume (PTV), prescribed isodose level (PIL) and treatment time (TT) for all clinical targets as well as for all phantom targets (see Eq. 5)

	Clinical targets	Phantom targets
β_0_	$9.23\cdot 10^{-1}$	$2.15\cdot 10^{-1}$
β_1_	$1.70\cdot 10^{-3}$	$1.34\cdot 10^{-3}$
β_2_	$1.45\cdot 10^{-5}$	$2.66\cdot 10^{-5}$
β_3_	$-2.13\cdot 10^{-5}$	$-1.30\cdot 10^{-5}$

Although there was no clear threshold, treatment plans with high conformity and low V12Gy tended to require a treatment time of ≥ 50 min. Treatment plans that showed a pronounced dependence of V12Gy on the isodose level were considerably shorter (≤ 45 min). In particular, planners should be aware that there is generally a strong trade-off between treatment time and dose gradient, as our results show. In our study, nCI, V12Gy, and treatment time were used to quantitatively characterize plan quality.

In general, in clinical situations, judgment about the quality and acceptability of a treatment plan will not be based solely on these three parameters. But V12Gy is highly correlated with other parameters as V10Gy (ρ > 0.99), V8Gy (ρ > 0.95), V4Gy (ρ > 0.53), GI (ρ > 0.81), and GM (ρ > 0.93) and thus may serve as a proxy for dose gradient and normal tissue toxicity, a view also supported by multiple SRS risk studies [32–37]. nCI is a measure of coverage and selectivity of the prescribed dose with respect to the target and was sufficient here as we only compared plans with identical coverage. Finally, treatment time was strongly correlated with two other metrics for plan quality, TotalMU (ρ > 0.94) and nBeams (ρ > 0.92). An increase in the TotalMU is associated with an increase in leakage radiation from the linac, although this should be balanced against the gain in improved dose gradient with increasing treatment duration. Short treatment time is mainly of practical advantage but has not been associated with improved clinical outcome [38].

### Limitations

Our study was limited to 4 spherical and 4 clinical targets with a volume ≤ 7.77 ml. This is a representative sample of the spectrum of brain metastases treated in robotic SRS, but whether our results also apply to larger or more complicated shaped targets, e.g., meningioma or vestibular schwannoma is left open. Also it would be interesting to study cases with multiple targets or to extend our study to other SRS techniques. Lastly, we only studied conical collimators and the Iris collimator, as the InCise multileaf collimator (MLC) of the Cyberknife is mainly used for stereotactic body radiation therapy [39]. Finally, our study focused on basic physical and dosimetric aspects of dose prescription in robotic SRS. We did not examine planning skills and abilities, which are known to vary widely [15, 40–42], but we were able to identify parameters and pitfalls that affect plan quality. Further, we did not examine the impact of the dose prescription level in SRS on clinical outcomes. However, a recent study provides evidence that inhomogeneous dose distributions may more beneficial than traditional ICRU-compliant homogeneous dose prescription in the treatment of brain metastases [17].

In our study, we analyzed the influence of PIL and collimator selection on dose homogeneity and plan quality for isocentric as well as non-isocentric irradiation techniques, for phantom as well as clinical targets, and with two different optimization algorithms. We did not aim to compare both algorithms and discuss their benefits or pitfalls, as this topic has already been presented in the literature [28]. Our results show that for the dose plans of both algorithms, there is a similar dependency between dose conformity, V12Gy, and treatment time on PIL and collimator selection.

## Conclusions

Using isocentric irradiation technique, selection of PILs is limited due to the size and shape of the beam profiles, whereas the achieved PIL is dependent on collimator sizes. In robotic SRS the non-isocentric irradiation technique allows the generation of highly conformal plans with steep dose gradients of inhomogeneous as well as homogeneous dose distributions with an intended PIL. Inhomogeneous dose distributions with a prescribed peripheral dose of ≤ 70% of the maximum dose showed the steepest dose gradients with simultaneous high dose escalation in the target and can be used to minimize toxicity to normal tissues. More homogeneous dose distributions within the target (up to 80% PIL) with a similarly steep dose gradient can be generated by using non-isocentric irradiation technique, careful selection of collimators and appropriate amount of treatment time. Generally, a combination of a mid-sized and a small collimator will be a good choice to achieve a desired PIL, steep dose gradient, and good conformity. PILs of ≥ 90% are associated with a marked and significant increase in off-target dose exposure, which must thoughtfully be traded off against the potential benefits of homogeneous on-target dose.

## Supplementary Information

Fig. S1–S5

Table S1: Results of the  isocentric irradiation of a phantom target with a volume of 0.27ml
Table S2: Collimator settings used in the non-isocentric irradiation plans of the four phantom targets and overview of all plan results of the Sequential optimization
Table S3: Collimator settings used in the non-isocentric irradiation plans of the four clinical targets and overview of all plan results of the Volo optimization

Tab. S2

Tab. S3
