# Supplementary material for: Impact of prescription isodose level and collimator selection on dose homogeneity and plan quality in robotic radiosurgery
Source: Strahlenther Onkol. 2021 Dec 9;198(5):484–96. doi: 10.1007/s00066-021-01872-4 (PMC9038902; doi:10.1007/s00066-021-01872-4)
Supplement: Supplementary file 1 — Fig. S1–S5 [file 66_2021_1872_MOESM1_ESM.docx]

**Supplementary Materials**

Figure S 1 Treatment plans of four patients treated with the Cyberknife for a single brain metastasis with a volume of (a) 0.50 ml, (b) 0.92 ml, (c) 2.01 ml and (d) 5.68 ml.

Figure S 2 Box plots of the volume of the 12 Gy isodose for three spherical targets with volumes (a,b) 0.55 ml, (c,d) 2.16 ml and (e,f) 7.77 ml as a function of the achieved prescription isodose level; (box plot representation of the smallest phantom target with a volume of 0.27 ml is missing, because there were too few data points per group.) Left column (a,c,e) shows data for all collimators. Right column (b,d,f) shows data where outlier curves (b: 7.5mm, 10mm, IRIS (5 .. 10mm); d: 12.5mm, 15mm; f: 20 mm) were excluded (see Figure 2d,f,h). P-values from the Wilcoxon rank-sum test are displayed at the top of each graph (*: p<0.05, **: p<0.01, ***: p<0.0001).

**

*Figure S 3* Box plots of the volume of the 12 Gy isodose for four single metastases with volumes of *(a,b)* 0.50 ml, *(c,d)* 0.92 ml, *(e,f)* 2.01 ml and *(g,h)* 5.68 ml as a function of the achieved prescription isodose level. Left column *(a,c,e,g)* shows data for all collimators. Right column *(b,d,f,h)* shows data where outlier curves (b: 7.5mm; d: 10mm; f: 10mm, 12.5mm; h: 12.5mm, 15mm) were excluded (see Figure 3b,d,f,h). P-values from the Wilcoxon rank-sum test are displayed at the top of each graph
(*: p<0.05, **: p<0.01, ***: p<0.0001).

**

*Figure S 4* Treatment time for four spherical targets with volume *(a)* 0.27 ml, *(b)* 0.55 ml, *(c)* 2.16 ml and *(d)* 7.77 ml as a function of the achieved prescription isodose level.

Figure S 5 Treatment time for four single metastases with volumes of (a) 0.50 ml, (b) 0.92 ml, (c) 2.01 ml and (d) 5.68 ml as a function of the achieved prescription isodose level.
